# Unusual Cutaneous Metastases From Colon Adenocarcinoma

**DOI:** 10.7759/cureus.14065

**Published:** 2021-03-23

**Authors:** Penélope Correia, Joana F Correia, Horácio Scigliano, Mário Nora

**Affiliations:** 1 General Surgery, Centro Hospitalar de Entre Douro e Vouga, Santa Maria da Feira, PRT; 2 Pathology, Centro Hospitalar de Entre Douro e Vouga, Santa Maria da Feira, PRT

**Keywords:** cutaneous metastasis, skin metastasis, colorectal adenocarcinoma

## Abstract

Skin metastases from internal malignancies are rare, even more from colorectal tumours, and are usually a sign of disseminated disease with a poor prognosis. However, with increased numbers of cancer survivors, a high index of suspicion should exist during the evaluation of cutaneous lesions since it can be the initial sign of disease progression or even the first sign that triggers a malignancy diagnosis, potentially improving the patient's odds. Herein, we report a case of synchronous cutaneous metastases from colon adenocarcinoma with unusual localization on the back and forearm of a 65-year-old man.

## Introduction

The skin is a relatively unusual site for internal tumour metastasis and remains a neglected, although relevant contributor, especially when total cancer incidence increases and more cancer survivors require surveillance for relapse [1]. Its prevalence stands around 1-4.3%, and major contributors from internal malignancies are breast and lung cancer, accounting for 32.7% and 13.2% of cases, respectively [1-6].

Although cutaneous metastases are usually a late manifestation of widely disseminated known malignant disease, in around 16-21% of cases they are the first sign of internal malignancy, as they are discovered before the underlying primary tumour [1,4,7]. 

Colorectal cancer (CRC) is one of the most frequently occurring cancers around the world, with 10% of them being diagnosed in stage IV [8]. The more common sites of metastasis are the liver, lung and central nervous system. Cutaneous metastases from colorectal cancer are extremely rare (4.2%) and indicative of poor prognosis, reflecting the spread of disease [1-3,6,9]. The most frequent sites of cutaneous metastasis are incisional scars at the abdominal wall, scalp, face and neck [4-5]. 

We present a case of synchronous cutaneous metastases from colon adenocarcinoma in an unusual localization.

## Case presentation

A 65-year-old Caucasian autonomous male without relevant past medical history presented to the emergency service in September 2019 with a one-month evolution history of worsening of his constitutional status, with a weight loss of 7 kg, associated with abdominal pain and distention with the sensation of reduced faecal transit. At clinical observation, he was thin, subicteric with a distended abdomen, which was slightly painful, without signs of peritoneal irritation. Analysis showed discreet leucocytosis and a cholestasis pattern with a total bilirubin of 2.94 mg/dL, lactate dehydrogenase of 510 U/L and C-reactive protein of 88 mg/L. Abdominal-pelvic CT-scan revealed multiple hepatic lesions suspicious of metastases, ascites and an irregular segmental wall thickening in the proximal sigmoid with permeable lumen and without imaging signs of occlusion.

**Figure 1:**
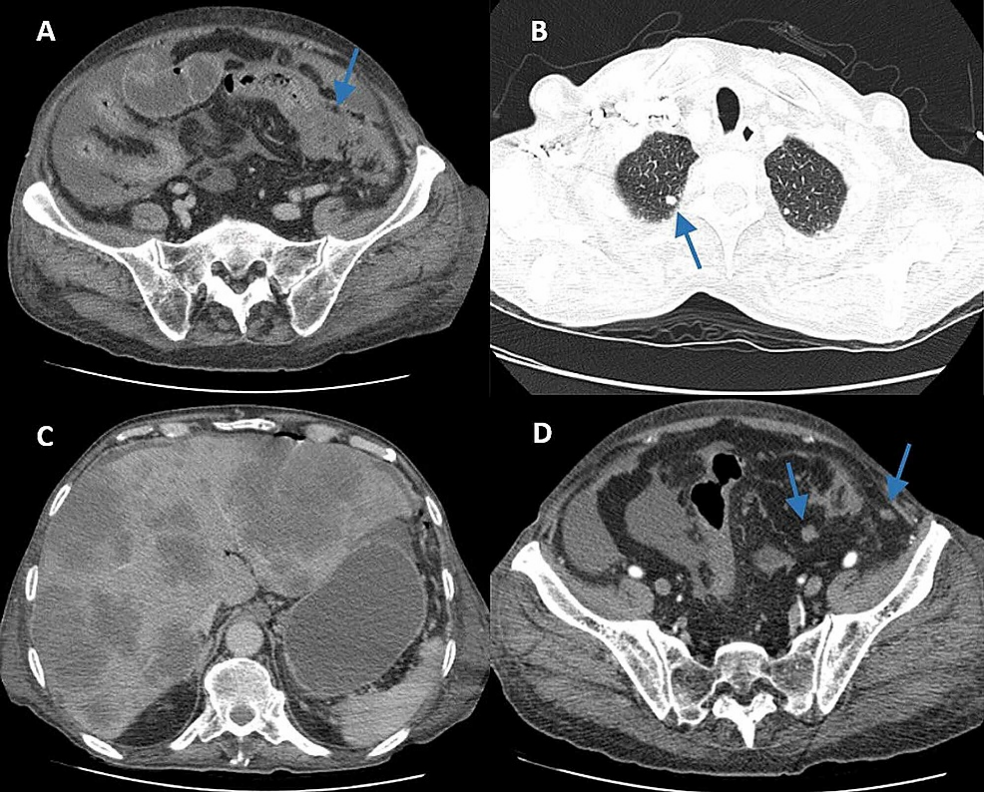
CT-scan images CT-scan images compatible with sigmoid neoplasia (A); pulmonary metastasis (B); liver metastasis (C); and peritoneal carcinomatosis (D)

**Figure 2:**
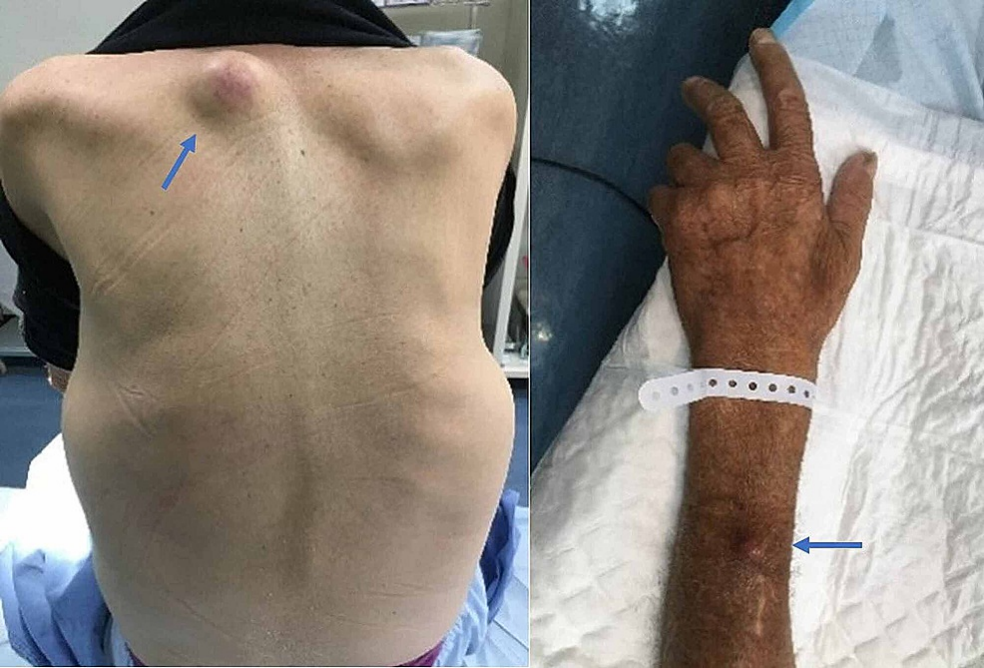
Nodular cutaneous lesions at the back and forearm (last one excised for analysis)

He was admitted for study and, from the performed exams, the presence of the following was noted: several nodules in the lungs and liver compatible with secondary lesions, moderate volume ascites, heterogeneous densification of peritoneal fat suggesting peritoneal carcinomatosis; irregular parietal thickening of proximal sigmoid colon, in relation to neoformative lesion; and multiple adenopathies (Figure 1). Carcinoembryonic antigen was recorded at >15000 μg/L. Rectosigmoidoscopy identified and biopsied the lesion at 35 cm of anal margin. We also identified two nodular, painless, hard and mobile cutaneous lesions, without ulceration: one at the back measuring approximately 4 cm and another at the forearm measuring 1.5 cm, and with a one-week evolution, that was excised for analysis (Figure 2). After clinical stabilization and pain control, the patient was discharged on the eighth day.

The patient died three days after discharge due to a rapid progression of disease and deterioration of his general condition.

**Figure 3:**
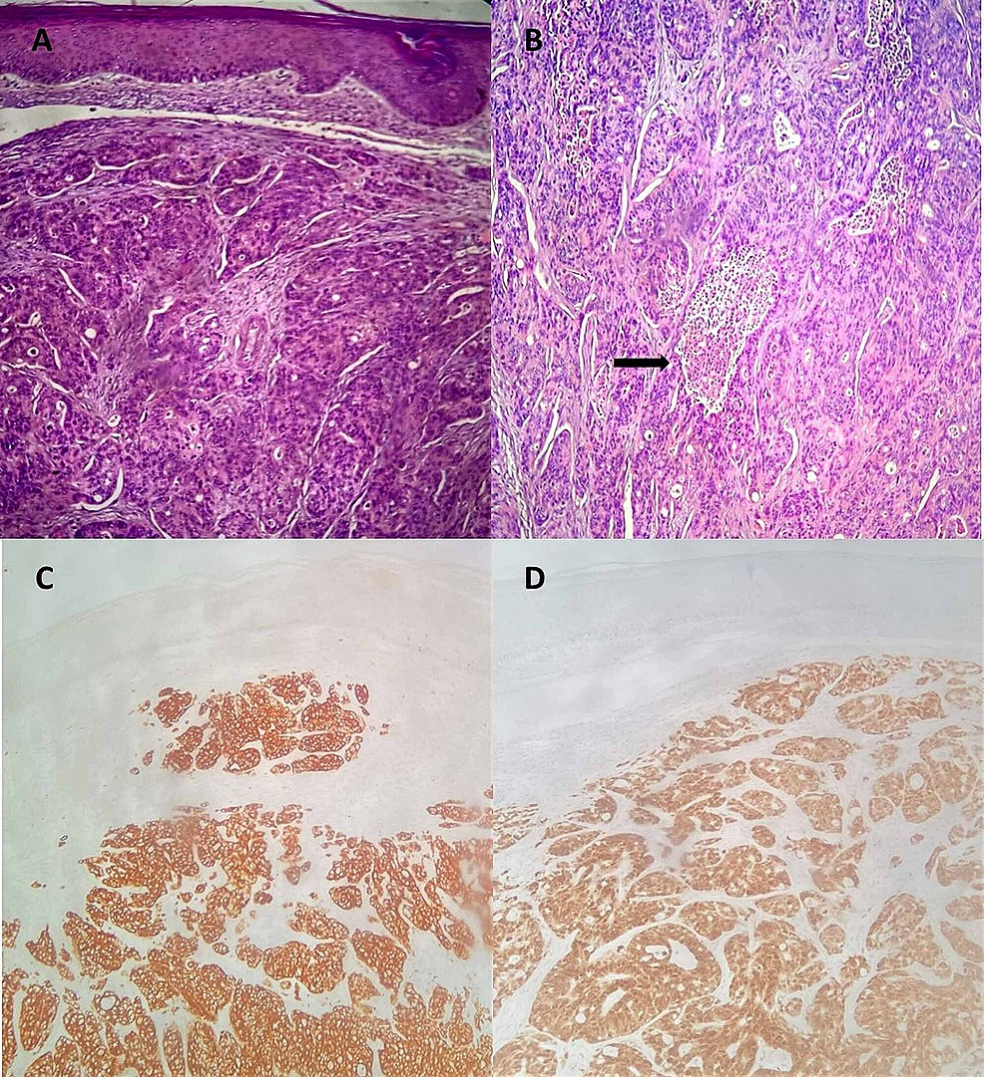
Metastatic colon adenocarcinoma involving the skin (A-D) Haematoxylin-eosin, original magnifications x 40 (A) and x 100 (B); C: cytokeratin 20; D: CDX2 (C-D - original magnifications x 40). Note in B the presence of typical dirty necrotic debris material with karyorrhectic nuclei (black arrow).

Histological results came after patient death: colon biopsy confirmed the presence of colon adenocarcinoma and excisional biopsy of the forearm cutaneous nodule proved to be a dermohypodermic metastasis of an adenocarcinoma with colic origin, with positive stain for CK20 and CDX2, and negative stain for CK7, Napsin A, GATA3, mammaglobin, ERG1 and PSA (Figure 3).

## Discussion

Cutaneous metastases are relatively rare, mainly from internal malignancies, in which prevalence is about 1-4.3%, the most frequent sources being breast and lung cancers [1-6]. Their occurence usually reflects a widely disseminated disease, associated with poor prognosis, with an expected survival ranging from 1 to 34 months [1-3,9].

Although CRC is one of the most common cancers worldwide, cutaneous metastasis from this kind of internal tumour is extremely unusual, occurring in 4.2% of all colorectal malignancies [1]. They typically occur within the first two years after primary tumour resection and are often associated with simultaneous metastasis in other organs [1-3,6,9]. However, cutaneous metastases can be the first sign of CRC, although less frequently.

The sites of cutaneous involvement are generally close to the primary tumour site, but distant metastases can also occur. For CRC, the face and scalp are the distant regions most commonly affected [4-5,7]. The patient described in our case showed an atypical localization for the emergence of metastasised cutaneous nodules, at the back, and even more unusually, the forearm.

The clinical appearance of cutaneous metastasis can assume various morphologies. It usually presents as painless, red/flesh-coloured mobile nodules, single or multiple, but they can mimic other cutaneous processes like lipomas, epidermal cyst, polyps, ulcers, cellulitis, zona zoster [1-2,7-8]. The ultimate diagnosis of cutaneous metastasis from CRC relies on histological evaluation. CK20, CDX2 and CK7 immunostaining are the most helpful markers to distinguish common malignancies, and CRC typically expresses CK20 and CDX2 but not CK7 [7-8,10].

The approach of primary tumour still is the step with the greatest impact on prognosis. Yet, interventions directed to cutaneous metastasis are showing increasingly relevant priorities for managing pain, tumour burden and quality of life, but no standard strategy has been defined [1-2,9-10]. Although conventional techniques like radiation and excision (when surgically feasible) remain the backbone of treatment [1,4,10], immunomodulatory therapies, such as electrochemotherapy and topic chemotherapy, have been showing hopeful results in cutaneous metastasis. However, stronger investigations and systematic studies are still needed [1,10].

## Conclusions

In conclusion, cutaneous metastasis from CRC is a rare phenomenon that usually signifies widespread disease associated with visceral metastasis, a sign of poor prognosis. Nevertheless, a high index of suspicion should be maintained in evaluating cutaneous lesions, particularly in patients with an oncologic past, since cutaneous metastasis could be the initial symptom of disease progression, or even be the first sign that prompts workup for malignancy, allowing for early therapeutic management and improving patient’s survival. Patient education also plays a major role in early detection and they should be familiarized with self-examinations, as often they would be the first to notice cutaneous alterations.
